# Assessing the Drying Sensitivity of Alkali-Activated Binders Through Mechanical Reliability: Effect of Particle Size and Packing

**DOI:** 10.3390/ma17225461

**Published:** 2024-11-08

**Authors:** Willian F. Camargo, Ana M. Segadães, Robinson C. D. Cruz

**Affiliations:** 1 Department of Engineering and Materials Science, University of Caxias do Sul, Caxias do Sul 95070-560, RS, Brazil; wfcamargo@ucs.br (W.F.C.); robinson.cruz@ucs.br (R.C.D.C.); 2 Department of Materials and Ceramics Engineering (CICECO), University of Aveiro, 3810-193 Aveiro, Portugal

**Keywords:** alkali-activation, blast-furnace slag (GGBFS), compressive strength, DoE, mechanical reliability, particle size distribution

## Abstract

Despite the steady progress of research on the alkali activation of wastes or subproducts from established industrial processes, the brittleness of the hardened alkali-activated materials frequently results in questionable mechanical reliability, particularly in industrial applications beyond construction materials. This work used a 3^3^ factorial Design of Experiments to examine the effect of three different particle size distributions on the compressive strength and mechanical reliability (Weibull modulus) of a sodium silicate-activated blast-furnace slag under the same processing conditions. As expected, curing temperature and time were strongly correlated, and the corresponding response surfaces showed that, for all studied particle sizes, compressive strengths above 60 MPa with mechanical reliability above 5.0 could be obtained by curing at ~60 °C for ~40 h. The particle size differences caused no significant changes in the extent of alkali activation, as seen in the infrared-spectroscopy results. However, the intersection of the response surfaces showed that a coarser and narrower particle size distribution extended the working area (time × temperature) and favored mechanical reliability. Thus, the precursor’s particle size distribution, which governs particle packing and viscosity during processing, also determines the permeability of the set binder, which affects water removal during drying and the dried binder’s mechanical performance.

## 1. Introduction

### 1.1. Inorganic Binders in Context

The last decades have seen a strong drive to reduce the environmental impact and carbon footprint of industrial processes, not only to ameliorate their harmful effects but also to appease consumers’ awareness of ecologically correct solutions. As an example, a plethora of papers can be found listing the environmental disadvantages of using Portland cement (PC) as structural construction material, compared with those of aluminosilicate-based alkali-activated binders (AABs). Claims mostly target high carbon dioxide emissions, the leading greenhouse effect culprit [1,2]. It should be noted; however, that such analyses seldom include the environmental footprint of producing the AAB ingredients, namely that of sodium silicate, which is the most used alkali-activator [3]. Nevertheless, in both cases (PC and AAB), the negative impact on the environment can be ameliorated by the use of alternative, frequently recycled, raw materials. The reuse of industrial wastes or subproducts from established processes does not add to the current harmful gas emission level and, thus, helps to avoid its increase. Moreover, it adds value to industrial waste that would otherwise be discarded or used in less noble contexts.

The mechanical and chemical properties of AABs are comparable to, or even better than those of ordinary cement [4,5,6,7,8,9] and, thus, they have become direct competitors in a variety of applications that demand high mechanical, chemical, and thermal performance, ranging far beyond the construction industry. On the other hand, in comparison with ordinary cement, the performance of AABs is more strongly dependent on processing parameters, and setting shrinkage can be too high [10,11], which might result in questionable mechanical reliability. Although this can, to some extent, be accepted in the construction industry, high-performance mechanical applications do not tolerate a large scatter in strength results within any given batch. Furthermore, if reliability issues can be waived, AABs might also stand as unexpected competitors for polymeric binders, or even metals, in such applications [12].

### 1.2. The Chemistry of Alkali-Activated Binders

Generally speaking, alkali activation is described as a complex process of structural rupture of the precursor and polycondensation of hydrated products into an aluminosilicate gel [9,13]. Thus, any material that contains a significant amount of reactive silica and alumina (preferably with an atomic ratio Si/Al > 1.5) and a given degree (preferably high) of structural disorder (i.e., amorphous or vitreous materials) can be used as a precursor in the formulation of AABs. Such precursors can be used either directly or after a thermal, chemical, or mechanical pre-treatment that enhances their reactivity [2].

The blast-furnace slag produced in iron extraction metallurgy, i.e., an existing subproduct, matches those required precursor characteristics. The fast cooling from the high-temperature metallurgical process results in amorphization and a vitreous structure. Due to the added fluxes and the impurities present in the iron ore, the slag can be particularly rich in calcium oxide (30 to 50 wt%), its other major chemical components being silica (28 to 38 wt%), alumina (8 to 24 wt%), and magnesium oxide (1 to 18 wt%) [7,14,15]. Thus, the blast-furnace slag has a chemical composition close to that of ordinary cement; its high calcium oxide content enables the formation of similar high-strength hydrated compounds, promoting early strength gains during hydration and presenting a similar setting behavior at low temperatures [2,16]. These characteristics explain why it is already abundantly used as a component in cement, as well as the potential of its use as a precursor in alkali-activated materials. However, certain reaction products come with an undesirable expansive formation, which can readily avert the mechanical strength gain and high durability [4,17].

References to the use of AABs based on blast-furnace slag as an alternative to common cement date back to the 1900s, namely in the patent registered by Kühl in 1908 [6]. Purdon suggested, in 1940, that alkaline hydroxides act as catalysts [18], and in 1980, Glukhovsky et al. identified the reaction products and described the simultaneous reactions in the alkali activation of a blast-furnace slag [19]. Since then, the research on alkali activation has grown exponentially, pursuing the development of high-performance binders [9,20].

Given that the curing process in alkali activation relies on chemical equilibrium, the process is commonly described as “chemical activation” [16]. On the other hand, cementing binders, similarly produced from highly reactive metakaolin, are specifically known as geopolymers [21]. During the calcination of kaolinitic clays (kaolin) between 500 and 750 °C, the de-hydroxylation of the structure occurs with crystallinity loss, and an amorphous aluminosilicate (metakaolin) is obtained [22]. Due to the kaolin high-temperature treatment, this process is described as “thermal activation”. Other processes, in which the precursor’s reactivity is promoted by the increase in surface area through comminution (grinding), with the decrease in particle size and incorporation of structural imperfections that might lead to amorphization, are described as “mechanical activation” [23].

The activator, whose role is to buffer the reaction pH at values basic enough to promote the dissolution of the aluminosilicate precursor [16], is generally a raw material with strong alkaline characteristics, such as sodium and potassium silicates or hydroxides, but it is far from limited to these two types of synthetic compounds [2]. Both are carbon intensive and present associated high handling hazards and high production costs [3]. The type of activator determines the behavior of the alkaline reaction and results in distinct properties after setting and curing. Compared to the use of liquid or powdered hydroxides, sodium silicate solutions are broadly used as activators, with claims that they promote compressive strength gain [24]. Commercial sodium silicates, with SiO_2_/Na_2_O weight ratios between 1.60 and 3.85, are usually produced at temperatures above 1350 °C by melting silica sand with sodium carbonate followed by dissolution in water in an autoclave, which explains their strong carbon footprint [2,3]. The concentrated alkaline solutions, thus, contain a complex mixture of silica oligomers in equilibrium with a population of isolated monomers, which are the most reactive. The solution’s viscosity, which contributes to the workability of the fresh binder, increases with the concentration and the SiO_2_/Na_2_O ratio [18]. The Si(OH)_4_ monomeric silica buffers the pH in the 11.0 to 13.5 range and guarantees a much higher alkalinity than that in hydroxide solutions [6]. When sodium silicate is used in the slag activation, a calcium silicate hydrate is also formed, which favors the cross-linking of the structure and promotes early mechanical strength. However, the mixtures lose flowability due to the hydrate’s fast formation, with a consequent workability loss.

Thus, understanding alkali activation begins with the molecular structure of the aluminosilicate-based precursors (silica tetrahedra and alumina octahedra linked by oxygen atoms) and their dissolution process [1,7,9,14,15,19,20,25]. Once the activator is added, the high hydroxyl concentration (OH^–^) forces the migration of positive ions (H^+^) from the precursor surface to the solution, thus enabling their replacement by the alkaline ions (Na^+^, K^+^) from the activator. The dissolution process then begins with the rupture of the Ca–O, Si–O–Si, Al–O–Al, Al–O–Si, and Mg–O bonds, increasing the Si and Al crowding in a layer that envelops the undissolved slag particle. When saturation is reached, dissolved colloidal phases precipitate as hydrated products, such as silanol (–Si–OH), sialates (–Si–O–), and gel-like amorphous phases from the network modifiers provided by the dissolving precursor, which then act as nucleation sites. In AABs with high calcium content, various reaction products that are typical in the Portland cement hydration also form, from calcium silicate hydrate (CSH) to the calcium aluminosilicate hydrate gel (CASH), or even, when magnesium, aluminum, and magnesium hydroxides, such as hydrotalcite, are present. Although free water is spent in this type of growth, diffusion processes dominate alkali activation and slow down the hydration reactions in the long run [10,13,26].

### 1.3. The Water in Alkali-Activated Binders

In terms of processing, the major difference between Portland cement binders and AABs is the curing agent: water in the first case and the alkali-activator in the second [6]. Ion exchange is essential in the precursor dissolution process, and thus, the presence of water is paramount, just as it is in common cement binders. However, common cement binding results from the formation of hydrated phases, which retain the water in the structure, whereas in AABs binding results from inorganic polymerization, the water molecules spent in the formation of Si(OH)_4_ and Al(OH)_4_ monomers are released later during polymerization [27]. In other words, despite the important role played by water in the ion exchange and dissolution kinetics, it does not notoriously take part in the formation of strongly hydrated cementing phases, such as those observed in Portland cement hydration. Therefore, the water remains in the set AAB microstructure as free water, which can be removed by spontaneous or forced drying above 100 °C, and as interstitial water, which can be removed between 150 and 300 °C [6,27].

Research in this area has been mostly aimed at investigating the effect of solid precursor’s load and the activator’s molarity on the final mechanical strength, paying little attention to the effect of water content on the curing phenomena or to the free water removal process. For instance, although the rise in curing temperature can lead to mechanical strength gains due to faster reactions [28], it can simultaneously encourage an abrupt capillarity water release, prompting internal microcrack formation that impairs the matrix structure. Curing time is also an important reaction parameter, as it affects both the reaction rate and the water release rate.

Moreover, the role of water during processing is of no lesser importance, namely during homogenization and in the shaping process (casting). Water can be introduced by the alkaline silicate (liquid fraction of the solution) or directly added to the mixture during homogenization. Both the mixing process and the mixture rheology during casting are expedited (i.e., decreased viscosity) by excessive water [29], but such excess has negative effects: lower water contents seem to promote higher mechanical strength and better chemical and physical stability, decreasing the danger of undesirable dimensional changes. Whatever its origin, the method used to finally remove the water can seriously affect the material’s microstructural characteristics and has a comparable, if not bigger, influence on the mechanical behavior of the cured binder.

During drying, because it is not chemically bound, the movement of the water on its way out of the body in response to the changes in capillary pressure (surface tension due to the water menisci) can cause non-uniform shrinkage and microstructural changes (e.g., micropores), which act as stress concentrators. Also, the matrix permeability provided by the porosity is usually not enough to accommodate the water vapor formed during drying, and the mismatch between formed and released vapor, dependent on the drying conditions, can induce stresses that surpass the material’s mechanical strength [27,30,31]. The ensuing crack formation, capable of resulting in catastrophic failure, is the major negative effect of the water removal on the mechanical strength [10,25,27]. For slag-based AABs, fast changes in drying humidity or sharp rises in temperature cause changes to pore structure and to the porosity due to free water, which can lead to microcracking and the resulting decrease in the AABs’ compressive strength [28,31]. Also, the calcium silicate hydrate (CSH) formation seems to be negatively affected when water evaporation is too fast. The comparison of infrared spectroscopy spectra (FTIR) of dried and undried AAB samples, used to study the structural changes caused by the drying method, suggests that the weight loss observed up to 120 °C is due to the evolution of both free and chemically bound water, while the weight loss due to the dehydration of hydrotalcite (aluminum and magnesium hydroxide) occurs only between 210 and 300 °C [5].

Thus, the development of porosity, which depends on the amount of water present during processing, also depends on how said water is removed from the set body. Crack formation can be slowed when fast drying is avoided, or drying is carried out under controlled humidity to keep the air close to saturation and ensure that water stays longer inside the drying binder [27]. Given the role played by the mixtures’ viscosity (rheology) in homogenization and casting, a decrease in added water might be compensated by the particle size distribution design [29,32]. This approach brings in yet another property balance, as an easier flow does not result in a more densely packed matrix [30]. However, by carefully choosing the precursor’s particle size distribution [33,34], it might be possible to manipulate (either ease up or hinder) the water removal “path”. That is, it might enable the control of the matrix interparticle porosity through particle packing and the development of a more permeable matrix, hence, less susceptible to mechanical damage caused by the water removal during drying. Bearing in mind the needed powder reactivity, which can be increased by mechanical activation by comminution, i.e., by the decrease in particle size with the consequent gain in specific surface area, a compromise between particle size distribution and particle fineness is likely needed to meet the desirable matrix characteristics [35].

This work aims to evaluate the role of precursor particle size and particle size distribution on the water removal during the drying of an alkali-activated matrix prepared from blast-furnace slag, sodium silicate solution, and water, under selected curing conditions (temperature and time) and its bearing on the mechanical reliability of the binder. To this aim, three different particle size distributions of the same blast-furnace slag (i.e., keeping constant the chemical composition) were used to prepare alkali-activated test-pieces under the same experimental conditions (i.e., processing parameters were also kept constant). Then, the mechanical reliability was assessed in terms of the Weibull distributions of compressive strength. Mercury porosimetry, thermogravimetry, and infra-red spectroscopy tests were used to clarify the effect of particle size on the water removal behavior.

## 2. Materials and Methods

### 2.1. Materials

A ground granulated blast-furnace slag (GGBFS) produced at Gerdau (Sapucaia do Sul, RS, Brazil) was used as a precursor. Three different particle size distributions were produced by grinding. The first, designated by “J”, was obtained by grinding in a jet mill (Jetmill S-Jet 25, Netzsch, Pomerode, SC, Brazil) for 30 min, at a classifier speed of 15,000 rpm and a product output of 1.2 kg/h. The second, designated by “B”, was obtained from the slag J by further grinding in a ball mill (MA500, Marconi, Piracicaba, SP, Brazil, total volume 5 dm^3^) for 8 h at 320 rpm with 80 % of the jar volume filled with alumina balls (4 kg of Ø32 mm spheres and 2 kg of Ø21 mm spheres). The third, designated by “J + B”, is a mixture of the two former ones in a 50:50 weight proportion.

The slag’s chemical analysis was carried out by X-ray fluorescence (XRF, EDX7000, Shimadzu, Kyoto, Japan). Its mineralogical analysis was carried out via X-ray diffraction (XRD, D8 Advance, CuKα radiation, Bruker, Ettlingen, Germany) between 10 and 100° 2θ, with a 0.05° step, at 4 s per step, at 15 rpm. Density was determined using Le Chatelier’s method in a calibrated volumetric flask.

The volume-based cumulative and frequency particle size distributions (PSD) of the three granulometries were obtained by laser diffraction (1090, CILAS, Orléans, France). The slag’s refractive index was taken as 1.6 [36]. The specific surface area was determined using the N_2_ adsorption BET method (NOVA 1200e, Quantachrome Instruments, Boynton Beach, FL, USA). Before the measurements, samples were degassed at 350 °C for 20 h, under vacuum.

The activator used in this work was a sodium silicate solution (PQ Solutions, São Paulo, SP, Brazil) with a SiO_2_/Na_2_O = 2.2 weight ratio. The solution’s density was determined using Le Chatelier’s method in a calibrated volumetric flask, the solids content was determined after drying at 180 °C until constant weight, and pH was measured with a pH meter (PMPH-2, Digimed, São Paulo, SP, Brazil). When needed, distilled water was used throughout the work.

### 2.2. Methods

To keep the viscosity of the fresh mixture of ingredients at values adequate for homogenization and test piece shaping by casting and, simultaneously, keep the processing parameters constant for all formulations, the slag and sodium silicate solution contents were set at 56 and 34 wt%, respectively, and that of the added water at 10 wt%. Mechanical stirring (propeller at 600 rpm) was used in the homogenization stage for 15 min. Viscosity was measured in a 200 cm^3^ volume of fresh paste using a spindle-type rotational viscometer (DV-II+ Pro, S64 spindle, Além Mar, São Paulo, SP, Brazil).

To examine the effects of curing parameters on the AAB compressive strength, a full 3^3^ factorial Design of Experiments was chosen. The literature shows ad nauseam that curing temperature and time significantly affect the mechanical performance of AABs, but their interaction with particle size has not been evaluated before. Given that, for systems in which temperature has a main effect, two-factor interactions involving temperature are likely to be observed, it is critical to use a design of experiments where calculations of the main effect are separated from the two-factor interactions (which rules out the use of fractional factorial designs) [37]. The three factors selected were slag granulometry (PSD), curing time, and curing temperature, each on three levels (Table 1), in a total of 27 configurations. To ensure the standardization of the binder’s characteristics, mandatory for the correct statistical treatment of compressive strengths, 40 test pieces were prepared for each of the 27 configurations. Once the mixtures were successfully prepared, the fresh pastes were cast into cylindrical polymeric molds (25 mm high and 12 mm in diameter) and left to cure inside the molds in the conditions described in Table 1 in a laboratory climatic chamber (MKFT 115, Binder, Tuttlingen, Germany) at 95% relative humidity.

After curing and test piece demolding, the compressive strength was measured using a universal testing machine (DL 2000/610, 2 ton loading cell, loading rate of 0.5 mm/min until failure, INSTRON-EMIC, São José dos Pinhais, PR, Brazil). Because these are ceramic-like brittle materials, the fracture behavior is expected to show a large scatter, even when the tested samples have identical physical–chemical characteristics. Such behavior is conveniently described by a Weibull distribution, which enables estimating failure probability within a large number of similar samples [38,39]. The Weibull modulus, m, is the distribution shape parameter, i.e., it represents the variability in fracture strength and allows the ranking of the material’s mechanical reliability (higher m values are synonyms of higher reliability). The Weibull mean strength (tmw) is calculated as the average of failure strengths weighed by the corresponding failure probability and the number of tested samples. In the Weibull fracture strength analysis, the cumulative probability function is written so that the failure probability, Pf, increases with the applied load. Equation (1) describes the failure probability of a test piece with a volume V, i.e., P_f_ = F(V), as a function of the applied stress, σ, and the Weibull characteristic strength, σ_0_, which is a scale parameter dependent on the test piece size and testing geometry [38,39].

(1) $$P_{f}=F\left(V\right)=1-exp\left[-{\left(\frac{\sigma }{\sigma_{0}}\right)}^{m}\right],$$

By taking the double logarithm of Equation (1), Equation (2) is obtained.

(2) $$ln\left\{ln\left[\frac{1}{1-F(V)}\right]\right\}=m\left(ln\sigma \right)-m\left(ln\sigma_{0}\right)=m\left(ln\sigma \right)-constant,$$

Thus, plotting ln(ln(1/(1 − F(V))) from a set of experimental results as a function of ln σ, is supposed to return a straight line whose slope is the Weibull modulus (or mechanical reliability), m. The Weibull characteristic strength, σ_0_, is the value of σ when the left side of Equation (2) is zero. In other words, σ_0_ corresponds to a failure probability P_f_ = 63.2%.

The same set of experimental results was also used to assess the correlation between factors in the Design of Experiments (Pareto charts and principal effects), i.e., to find out which factor combination (time, temperature, and particle size) had the strongest effect on mechanical reliability [37]. The mechanical reliability (m) and the Weibull mean strength (tmw) were plotted as response surfaces for each particle size distribution (J, B, J + B) to better observe the interaction between curing temperature and time. The software Minitab 22.1.0.0 was used to construct the response surfaces.

Based on the response surface analysis, the combination of temperature and time for the best mechanical performance was chosen. Ten new test pieces for each granulometry were then prepared to investigate the changes in density and residual water contents (weight loss relative to total, theoretical, and weight loss) during curing at the selected temperature (oven with no humidity control). To ensure that test pieces had the strength necessary for handling and property measurement, the heat treatment was subdivided to include a pre-cure stage, which was considered as a reference for subsequent measurements: after casting, the filled molds were kept at 30 °C for 24 h (pre-cure); then, the test pieces were demolded, and the heat treatment continued at the prescribed temperature, measurements being taken every 2 h until completing the prescribed treatment time. Values were calculated from test piece weight and dimensions. The final macrostructure of the heat-treated bodies was observed with an optical stereo-zoom microscope (M7A Wild-Heerbrugg, Gais, Switzerland).

After pre-cure and at the end of the heat treatment, pore sizes were measured by mercury intrusion porosimetry (Pore Master, Anton Paar, Boynton Beach, FL, USA) from 1060 µm to 3.6 nm, corresponding, respectively, to filling volumes of 0.5 to 2 cm^3^ at the maximum intrusion pressure (0.2–50 psi, i.e., 1.4–345 kPa). Fourier transform infrared spectroscopy (FTIR) was carried out with a Nicolet iS10 (Thermo Scientific, Waltham, MA, USA), scanning wavelengths from 500 to 4000 cm^−1^, with 0.5 cm^−1^ maximum resolution. Thermogravimetric analyses were carried out (STA 449 F3 Jupiter, Netzsch, Selb, Germany, at a heating rate of 10 °C/min up to 400 °C) using ~20 mg of binder sample in a platinum crucible and synthetic air (40 cm^3^/min) as purging gas.

## 3. Results and Discussion

### 3.1. Characterization of Activator and Precursors

The sodium silicate solution used as the activator (weight ratio SiO_2_/Na_2_O = 2.2) has a density of 1.48 g/cm^3^ and a solids’ content of 49.4 ± 1.0 wt%. The blast-furnace slag is a grayish-beige powder of fine granulometry, with a density of 2.78 g/cm^3^. Its chemical composition (major oxides) is presented in Table 2. The calcium oxide content is comparatively low, its other major components being silica, alumina, and magnesium oxide, as expected.

**Figure 1:**
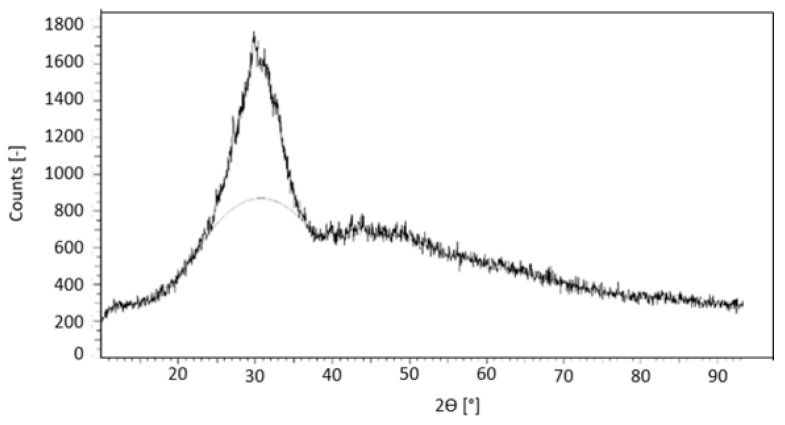
X-Ray diffractogram of the as-received blast-furnace slag.

Also, as expected, the slag’s X-Ray diffractogram (Figure 1) presents the background hump characteristic of an amorphous material. The amorphization degree is approximately 68%. The broad, well-pronounced peak around 30° 2θ reflects the short-range order within the CaO–Al_2_O_3_–MgO–SiO_2_ structures. Both the amorphous character and the atomic ratio Si/Al = 1.064 show that the slag is adequate to be used as a precursor.

**Figure 2:**
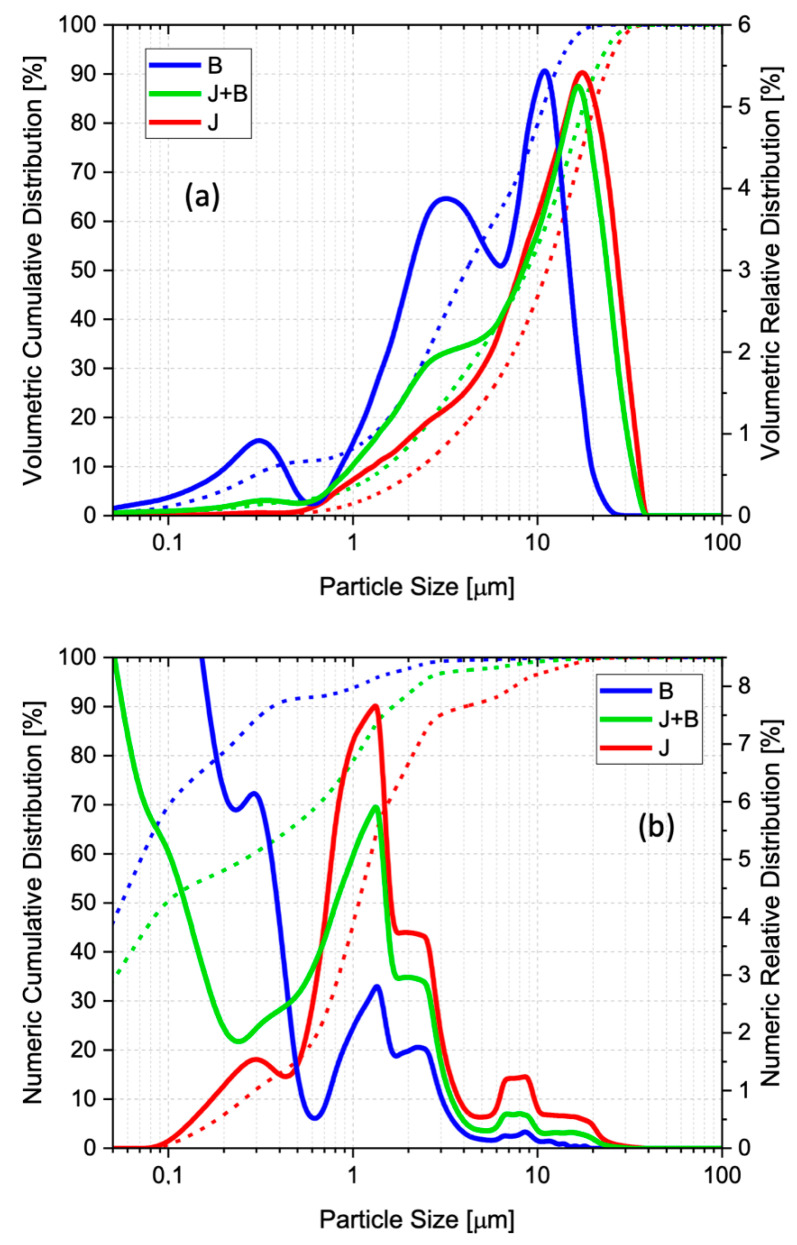
Volume-based (**a**) and number-based (**b**) frequency particle size distributions for samples J, B, and J + B (dashed lines represent the cumulative distributions).

High mechanical strength is generally associated with dense (less porous) matrices produced from high reactivity particles (i.e., fine particles with high specific surface area). Particle packing efficiency is directly related to the particle size distribution (PSD). The cumulative and frequency particle size distributions of the three slag granulometries used, expressed on a volume and number basis, are shown in Figure 2.

For the sample obtained from the jet mill (J), the expected low dispersion volume particle distribution (unimodal) can be observed, as the size classifying system in the mill separates the particles whose size lies outside the range defined by the grinding parameters. The distribution curve is narrow (median size d50 = 11 µm), and there is a low volume of particles < 0.1 µm. The subsequent ball mill grinding (B) resulted in a polydisperse volume distribution of smaller sizes, with peaks (modes) at three different sizes: ~0.3 µm, ~3 µm, and ~9 μm. According to the literature [32], the packing density of a material with a broad particle size distribution (bi- or multimodal) is higher than that of the same material with a narrower PSD (monomodal). Thus, one would expect that particle packing in B will be better (denser) than in J. The additional grinding decreased d50 from 11 to 4 µm and generated an increased number of fine particles (95% < 1 µm). The number-based distribution shows that the majority of particles are concentrated at the ~0.3 μm mode seen on the volumetric curve, which loses importance in the volume-based distribution curve, i.e., the massive quantity of fines present only becomes apparent when the PSD is expressed on a number base [40].

Both volume- and number-based size distributions for J + B lie between the other two, as expected for a 50/50 mixture. As mentioned before, volume-based distributions are particularly sensitive to larger particles [40], which in this case come from sample J. Thus, the volume d50 for sample J + B (9 µm) is close to that of J (11 µm). The finer fraction effect is better seen on the number-based cumulative distribution curves and, in number, J + B’s d50 (100 nm) is much closer to that of B (60 nm) than to that of J (1.1 µm).

The multi-modality nature of a particle size distribution can be characterized by the distribution’s span (width), that is, the distance between two points equally spaced from the median d50, commonly d10 and d90, relative to that median [41]. The values obtained for the span were 1.85 for J, 2.14 for J + B, and 2.92 for B. Thus, not only is the median size higher for J (d50 = 11 µm), but also an increase can be seen in the multi-modality level relative to J, of 16% for J + B (d50 = 9 µm), and of 58% for B (d50 = 4 µm).

The specific surface area values obtained for J and B were 2.33 and 2.81 m^2^/g, respectively. This confirms the decrease in particle sizes brought in by the extra ball mill grinding, as seen in the volume-based particle size distribution in Figure 2. The finer fraction that explains the surface area gain will also promote additional surface reactivity.

### 3.2. Choosing Homogenization and Casting Processing Parameters

The literature suggests an activator/precursor weight ratio of 0.4 to meet the cation ratios ideal for best performance after curing [28]. However, besides particle packing, differences in particle size and particle size distribution also have some bearing on the suspension’s viscosity, i.e., on processing, namely on homogenization and shaping by casting [29]. Thus, to reach a suitable viscosity value while keeping the processing parameters for all formulations constant, the activator/precursor proportion as well as the added water content, had to be adjusted. Prior testing showed that mixing times (600 rpm) shorter than 15 min resulted in heterogeneous mixtures, whereas mixing for longer than 15 min trapped persistent air bubbles into the mixture. Prior testing also showed that, for those stirring parameters, there is an ideal viscosity range: viscosities below 1500 cP (1.5 Pa.s) promoted the imprisonment of a great volume of bubbles (generally meant a low solids’ content), thus resulting in cured bodies with low mechanical strength; viscosities above 4500 cP (4.5 Pa.s) impaired the shaping of test pieces with comparable characteristics (generally meant an excessive solids’ content and accelerated curing reactions). Thus, for the chosen stirring conditions and all the tested particle sizes, the mixtures containing 56 wt% slag, 34 wt% sodium silicate solution, and 10 wt% added water, presented a viscosity close to 3000 cP (3 Pa.s), which ensured the uniformity of shaping as well as the characteristics of the resulting test pieces. Therefore, all formulations initially contained 27.2 wt% water.

The literature shows that, for a constant solids’ content [32], as in this work, the suspension’s viscosity increases when the particle size decreases (the larger number of fine particles results in more interactions among them, hence, a higher flow resistance). This alone would suggest that a lower viscosity should be expected for J, which has the highest d50, and a higher viscosity for B, which has the lowest d50. However, the literature also shows that, for that constant solids’ content, a multimodal PSD results in a viscosity lower than that of both monomodal suspensions with only coarse or fine particles [32]. The preliminary tests carried out in this work to define the processing parameters soon showed that the B suspension presented the lowest viscosity, its stirring being more turbulent and prone to entrapping a larger volume of air bubbles, which shows that the multi-modality effect on the suspension’s viscosity outweighs the effect of the particle size.

Given that all formulations have the same proportion of ingredients, the mixtures’ initial density was calculated as a weighted average of the densities of the individual components. Thus, by using the corresponding density values (i.e., 2.78 g/cm^3^ for the slag, 1.48 g/cm^3^ for the sodium silicate solution, and 0.99 g/cm^3^ for water) and weight fractions in the formulation (i.e., 0.56 for the slag, 0.34 for the sodium silicate solution, and 0.10 for water), the initial density was 2.15 g/cm^3^. Therefore, in chemical terms, AABs had the atomic ratios Si/Al = 1.71 and Al/Na = 1.75 and were prepared with a weight ratio H_2_O/Na_2_O = 5.18.

### 3.3. Mechanical Characterization of AABs

**Figure 3:**
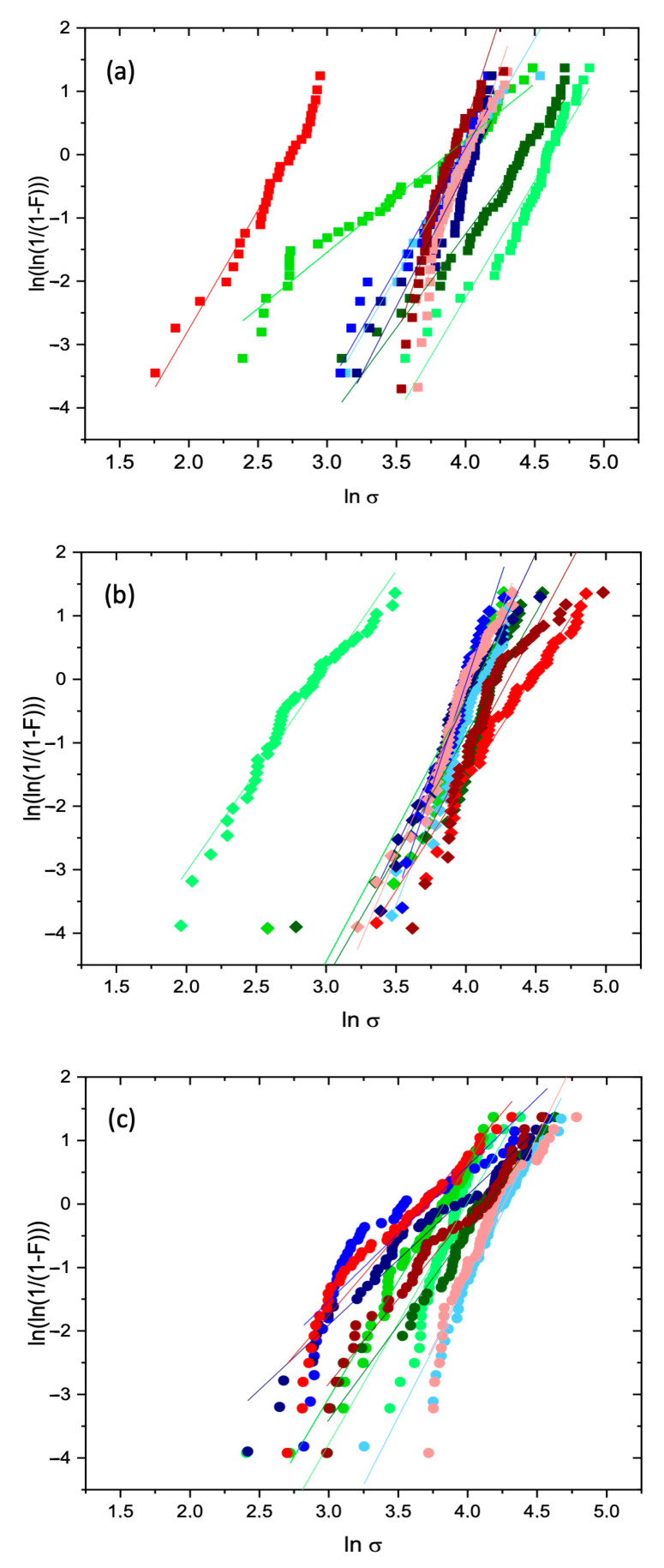
Compressive strength Weibull distributions for the AABs J, B, and J + B cured at: (**a**) 30 °C; (**b**) 60 °C; (**c**) 90 °C. The symbol and color legends are presented in Table 3.

Table 3 shows the values for the Weibull mean strength (tmw), Weibull modulus (m), and the R^2^ for each linear regression for all 27 sets of test pieces. For the sake of clarity, the results are grouped by curing temperature. The compressive strength of Weibull distributions used to determine the values of m are shown in Figure 3. The symbol and color legends for Figure 3 are shown in Table 3. In Table 3, the R^2^ value obtained for the linear regression fitting to the experimental results confirms that the test pieces were consistently produced and that the tests were correctly carried out. The swarming of the experimental results close to the regression lines in Figure 3 suggests the presence of a dominant single defect type.

From the point of view of mechanical reliability, the generally low values obtained for the modulus m (slope of the regression line) confirm that the material is brittle and, as such, of reduced reliability. On the other hand, high mean strength values (tmw), although desirable, are no strong assurance of the matrix integrity. A given set of parameters can, at times, result in above-average compressive strength, and within the same Weibull distribution, lower-than-expected compressive strength values might still occur. This would broaden the strength distribution and decrease the mechanical reliability.

The average of all the compressive strength values is 53.65 MPa. This average is in good agreement with the literature, which reports values in the range of 20 to 45 MPa for cured slag-based AABs [42,43]. The highest tmw value was 91.39 MPa (found for the configuration “J, 30 °C, 24 h”), i.e., 70% above average. However, the corresponding mechanical reliability was 3.49, that is, 12% below the average of 3.93. Granulometry B showed the lowest mechanical strength values in nearly all curing conditions, generally coupled with, also low reliability values.

These findings clearly show that the production of an AAB matrix with high structural integrity under compression involves a compromise between m and tmw. In Table 3, the five highest Weibull mean strength values (tmw) and Weibull modulus (m) are highlighted in bold. It can readily be seen that there is no clear correlation between m and tmw maxima; other than that, they most frequently occur for the curing temperature of 60 °C and the granulometry J.

**Figure 4:**
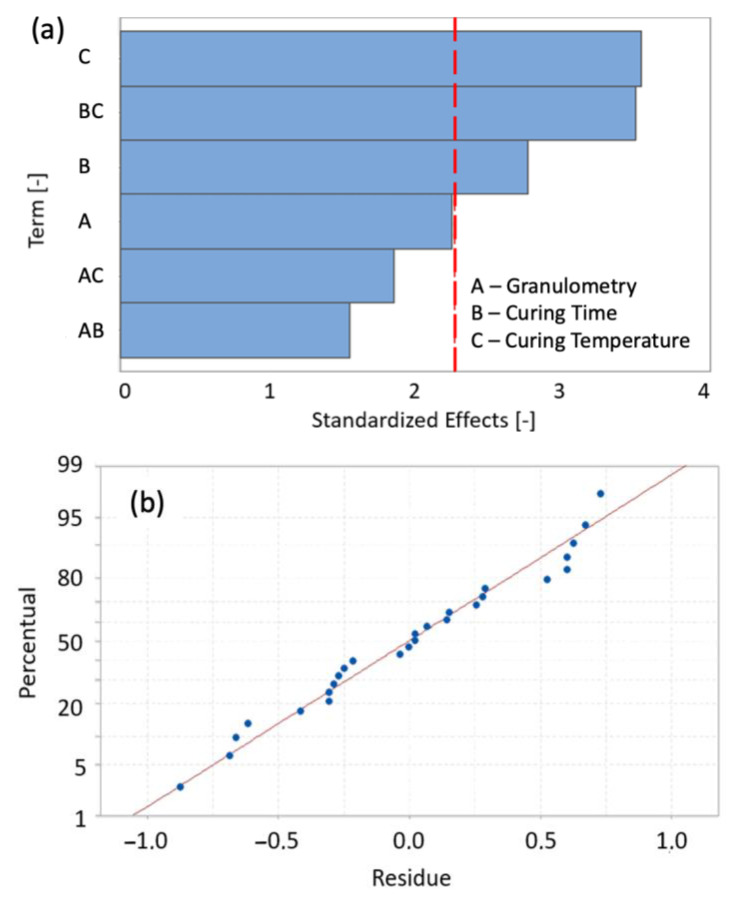
Pareto chart of standardized effects on mechanical reliability for a significance level of 0.05 (**a**) and normal probability plot (**b**), using granulometry, curing time, and curing temperature as factors [37].

To find out which factors have the most relevant effect on the AABs’ mechanical reliability, a correlation analysis was carried out, and the Pareto chart of standardized effects was constructed for a 0.05 significance level (Figure 4a). In such a graph, horizontal bars represent the magnitude of each effect, and the vertical line going across the bars indicates the chosen significance level. Only the effects whose bar is longer than the vertical line are statistically significant. The normal probability plot in Figure 4b shows that the residuals are linearly related to the expected normal values. Therefore, the statistical analysis can be considered valid [37].

As expected for a “standard recipe”, the curing temperature (C) and the combination of curing temperature and time (BC) have the most significant effect on the AABs’ mechanical reliability. However, Figure 4 also shows that curing time (B) is important, too, immediately followed by granulometry (A). Interestingly, though, in combination with any other factor, the granulometry appears to affect the mechanical reliability to a rather lesser extent than the other factors.

**Figure 5:**
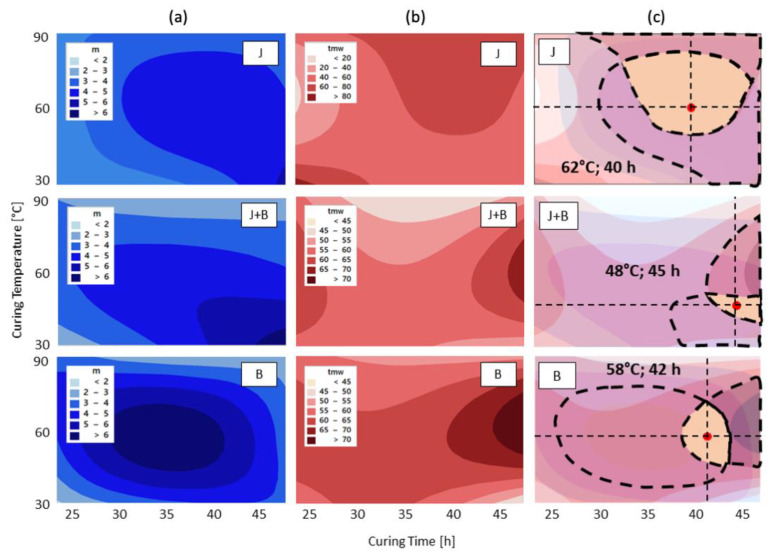
Iso-property contour plots as a function of curing time and temperature, for the granulometries shown: iso-mechanical reliability, m (column (**a**)), iso-Weibull mean strength, tmw (column (**b**)), and overlapped contour plots for iso-m and iso-tmw (column (**c**)) highlighting the favorable working area.

Based on the factor correlation analysis (Figure 4), the response surfaces for mechanical reliability (m) and Weibull mean strength (tmw) were constructed for each of the granulometries as a function of curing time and curing temperature. Such response surfaces are presented in Figure 5 as iso-property contour plots (column (a) for m and column (b) for tmw). This type of graphical representation shows with enhanced clarity that high temperatures or very short curing times are detrimental to both m and tmw. The areas with higher values suggest that the best combination is, in all cases, extended time at moderate temperatures.

For each granulometry, J, J + B, and B, the overlapping of the corresponding iso-m and iso-tmw contour plots (column (c) in Figure 5) brings to evidence the working area (i.e., time and temperature combinations) that guarantees a response with a specified minimum property value [44]. Thus, for the granulometries investigated in this work, to ensure a compressive strength tmw above 60 MPa with a reliability m of at least 5.0 while keeping constant all processing parameters, it can be said that the most promising curing conditions to equally suit all granulometries, will be close to 60 °C for the curing temperature and 40 h for the curing time. It can be seen that J is the most forgiving granulometry, i.e., it offers the broadest working area. Although the working area for J + B is a little distant from the working areas of the other two, this exercise demonstrates the expected prevalence of the joint effect of temperature and time for a given set of constant processing parameters. Furthermore, it emphasizes the subtle effect that particle size has on the extent of the working area, particularly so for mechanical reliability.

### 3.4. Water Removal and Its Bearing on the Mechanical Reliability of AABs

**Figure 6:**
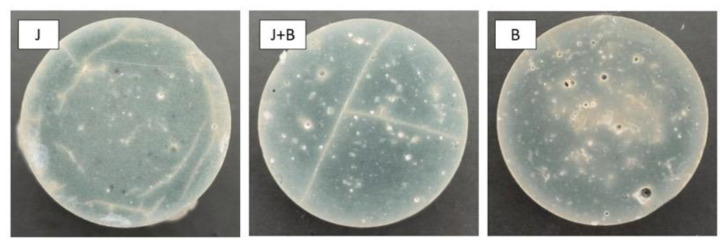
Typical macrostructure of the final heat-treated bodies.

**Figure 7:**
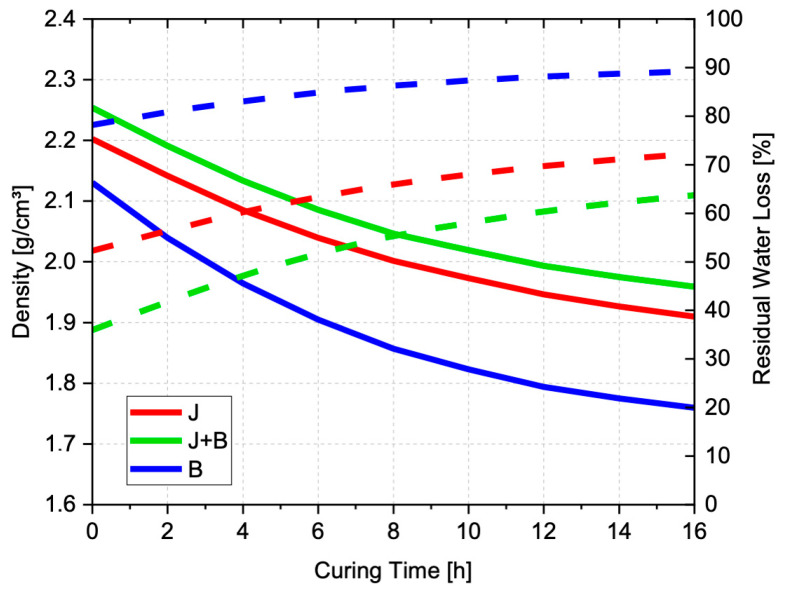
Changes in the test pieces’ density during the cure at 60 °C for up to 16 h (continuous lines) and the corresponding residual water variation (dashed lines).

Based on the mechanical characterization results, new test pieces were prepared for the three granulometries, to evaluate how the body density changes during the cure and, thus, shedding some light on the water removal process. After the pre-cure (24 h at 30 °C), samples were demolded, measured, and weighted, to calculate the density and residual water at zero time. The residual water is what is left from the initial water used in the AAB preparation (i.e., 27.2 wt%), which remains in the test piece and is progressively removed during the curing process. Figure 6 illustrates the typical macrostructure of the final heat-treated bodies, and Figure 7 shows the changes in density and residual water, as measured every 2 h during the ensuing cure at 60 °C.

As all formulations have the same composition and suspension density (calculated as 2.15 g/cm^3^), the final cured and dried bodies’ density should be 1.57 g/cm^3^ at least, assuming complete water elimination and zero shrinkage. After the pre-cure (time t = 0) and demolding; however, some diametral shrinkage could be observed (3.82, 4.31, and 5.75%, respectively for J, J + B, and B), as well as pronounced weight loss, which is basically due to water removal. Moreover, the cured bodies presented different densities: 2.20, 2.25, and 2.13 g/cm^3^, respectively, for J, J + B, and B). Cured bodies produced with the finer granulometry, B, which were expected to show the highest density values at pre-cure, presented the lowest density and largest shrinkage and weight loss. The suspension prepared with B showed a viscosity lower than those of the suspensions prepared with the coarser powders because, as mentioned before, the effect of the corresponding multimodal PSD on the viscosity seems to overcome that of the finer particle size [32]. As a result, the air entrapped during stirring of the low viscosity suspension is in some way retained during the pre-cure and remains in the set structure as porosity (Figure 6). Thus, this added porosity might explain not only the lower pre-cure density but also the easier water loss (78.2% of the initial water is already lost at pre-cure), and the subsequent weaker mechanical performance of these compositions (Table 3). Said mechanical performance does not worsen even further only because the higher surface reactivity of the finer particles accelerates the chemical reactions, and water ceases to be needed sooner [27].

As for J and J + B, their initial densities are higher and closer to each other, just as generally observed for the corresponding mechanical strengths. As expected, the lower packing efficiency of the particles in J, as compared to J + B, clearly results in lower density (2.20 as opposed to 2.25 g/cm^3^) and less residual water (52.3 as opposed to 35.9% of the initial water is lost at pre-cure), i.e., higher porosity and permeability. Nevertheless, the corresponding final microstructure might contain fewer (or smaller) microdefects than that of the J + B bodies, since, as seen in Table 3, the mechanical strength of the latter tends to be consistently lower.

The effect of the ensuing cure at 60 °C is, for all granulometries, no more than a continuous and smooth decrease in the density with time, towards the calculated theoretical value (1.57 g/cm^3^), without significant dimensional change. This suggests that the final structure is irrevocably defined during the pre-cure, in response to the specific particle packing (i.e., porosity). In other words, the pre-cured bodies no longer accommodate dimensional changes to relieve internal stresses induced by the subsequent water removal, i.e., they are already stiff and, therefore, mechanically brittle, which might explain the catastrophic results of aggressive cures.

**Figure 8:**
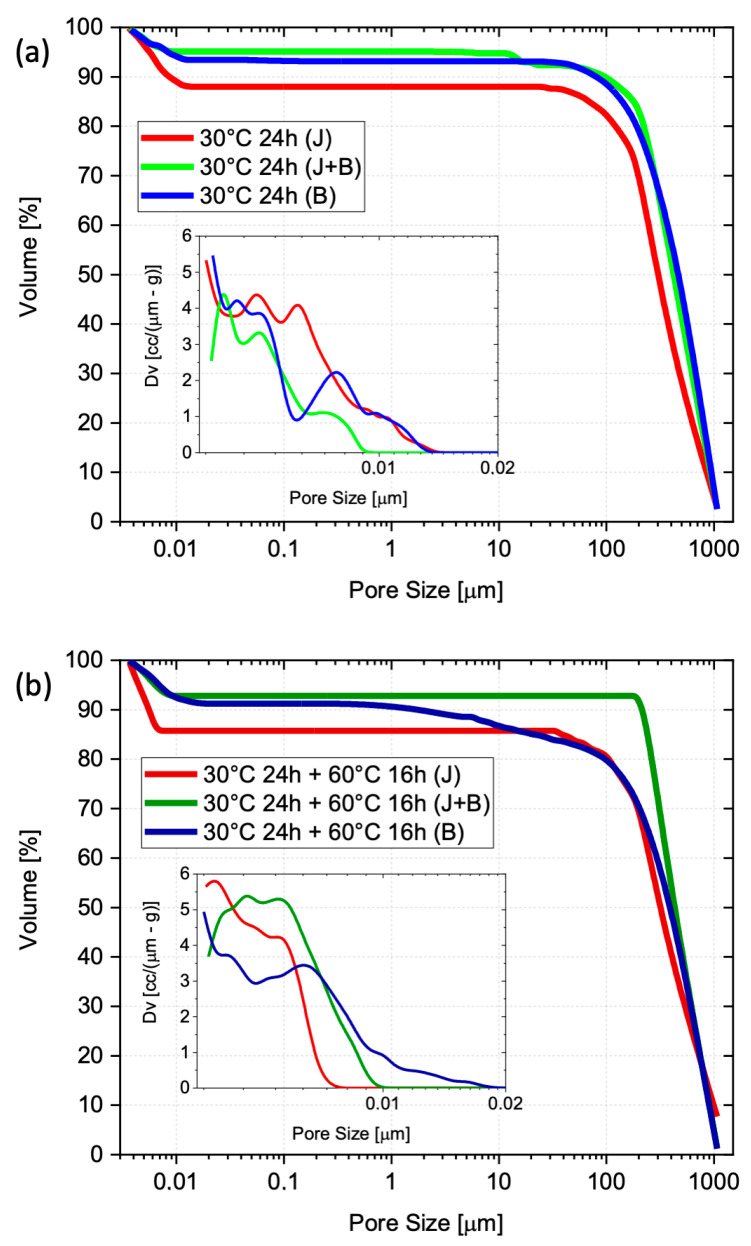
Cumulative pore size distribution (vol%) in the AAB test pieces: (**a**) after the pre-cure (24 h at 30 °C), and (**b**) at the end of the heat treatment (16 h at 60 °C). The insets show the corresponding frequency pore size distribution (vol%).

The cumulative pore size distributions were determined by mercury intrusion porosimetry, after the pre-cure and at the end of the subsequent heat treatment at 60 °C for 16 h (Figure 8). For all formulations, it can be seen that there is no significant difference between before and after the cure at 60 °C, with the majority of pores having sizes below 200 µm (the cumulative curves level out just below 90 vol%). This confirms that the pore distribution, hence the AAB structure, is mostly defined during pre-cure. For the J samples, the majority of pores are below 10 nm, whereas only B samples show two dominant pore sizes (insets in Figure 8).

From the particle packing point of view, these findings suggest that a coarser and more uniform granulometry (i.e., J), with lower packing efficiency, might be favorable during the alkali-activation reactions and the subsequent water removal stage. The small size of most pores (i.e., small defect size) benefits the AAB’s compressive strength. For the granulometries containing a relevant fine particle fraction (i.e., J + B and B), the observed presence of larger pores (>1 µm) might be related to the air bubbles entrapped during stirring, as seen in Figure 6 and already mentioned while discussing the density changes shown in Figure 7.

The analysis of the pore size frequency distribution curves shown in the insets in Figure 8, which highlight the sizes below 30 nm, also supports this argument. Being a volume-based distribution, a sheer number of pores is needed to generate peaks in the nano-size region. Thus, all six curves suggest that the number of nanopores in all cured bodies must be various orders of magnitude higher than that of larger pores.

**Figure 9:**
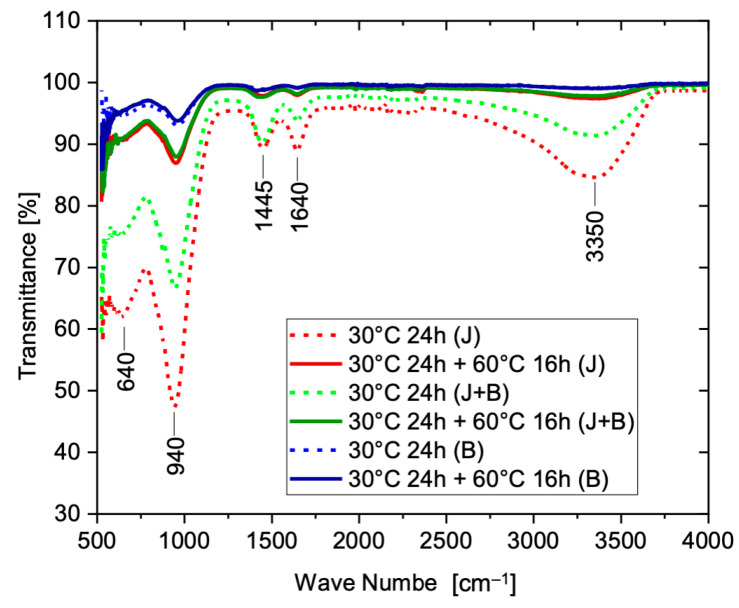
Infrared spectra (FTIR) obtained for the AAB test pieces after the pre-cure (24 h at 30 °C, dashed lines) and at the end of the heat treatment (16 h at 60 °C, continuous lines).

The infrared spectroscopy analysis (FTIR) helps to identify, through the most frequent bond vibration bands, the alkali-activation reaction products and the presence of free water. Figure 9 shows the FTIR spectra obtained for the AABs after pre-cure and at the end of the heat treatment. According to the generally accepted literature, the alkali- activation reactions frequently exhibit bands near 1000 cm^−1^, attributed to the Si–O–Al and Si–O–Si asymmetric stretching vibration. These bands tend to shift to lower frequencies and decrease in intensity when the polymerization degree increases. The Si–O–Al and Si–O–Si bending vibration bands occur between 650 and 600 cm^−1^. A broad band centered at 3300 cm^−1^ corresponds to the stretching vibration of the O–H bonds in water, both free (higher frequencies) and involved in hydrogen bonds (lower frequencies). It might also be related to the non-crystalline character of the precursor. A weak band between 1600 and 1700 cm^−1^ represents the H–O–H bending vibration of adsorbed H_2_O molecules. Another weak band between 1300 and 1500 cm^−1^ signals the presence of sodium carbonate formed by carbonation during the cure but is also related to the Si–O–Al asymmetric stretching vibration [45,46,47]. As the alkali activation progresses, the matrix structure becomes increasingly stiff, and the bands become less intense (reduced interaction between bonds and infrared light wave).

In Figure 9, the curves relative to the pre-cured bodies (dashed lines) show deeper bands at 940 cm^−1^, typical of an incomplete reaction, and in the characteristic of a water vibration range near 3000 cm^−1^. However, the FTIR spectrum for the finer granulometry (B) already shows considerable reaction progress and reduced intensity in the characteristic water bands, thus supporting the lower residual water content seen in Figure 7. The Si–O–Si bands are also less intense, showing how the increased precursor’s surface reactivity due to the extra grinding accelerates the binder’s reaction.

A comparable reaction level is only reached by the other two granulometries after the complete heat treatment (24 h at 30 °C + 16 h at 60 °C). For the finer granulometry, the full treatment curve practically coincides with the corresponding pre-cure curve. The porosity differences could also lead to band shifts but the heat treatment after the pre-cure seems to be the major cause of the changes in the band intensity.

**Figure 10:**
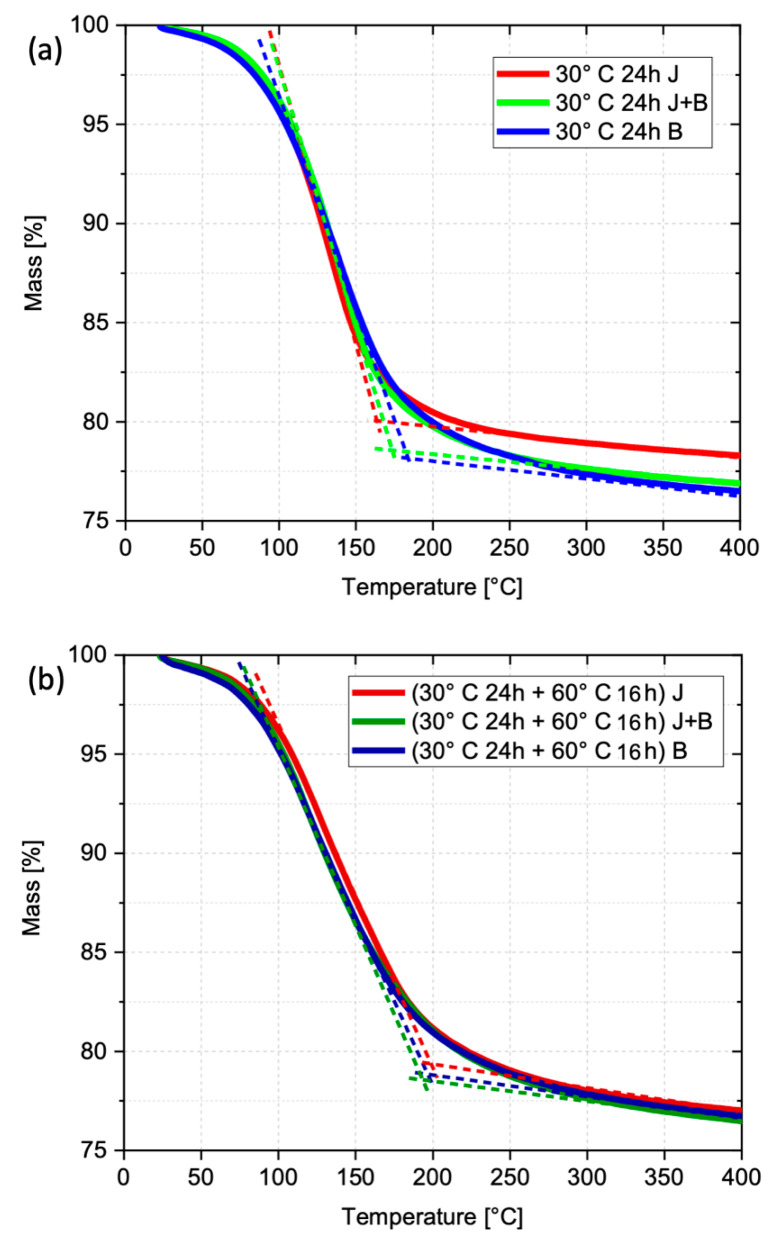
Thermogravimetric analysis of the AAB test pieces: (**a**) after the pre-cure (24 h at 30 °C) and (**b**) at the end of the heat treatment (16 h at 60 °C). Dashed lines signal the end of the weight loss (offset).

The thermogravimetric analysis (Figure 10), either before or after the cure at 60 °C, shows weight losses exclusively due to water removal, given that the AABs are chemically stable under the used testing conditions and do not release any other volatile compounds below 400 °C. No significant differences can be observed among the various granulometries, neither after the pre-cure nor at the end of the heat treatment at 60 °C. Nevertheless, as shown by the curves’ offsets, the pre-cured bodies (24 h at 30 °C) tend to lose practically all the physically bonded water earlier (below 175 °C), as compared to those with the complete cure (closer to 200 °C). These results confirm those obtained by FTIR, suggesting that alkali-activation reactions progress, albeit slowly, during drying. The full-cured bodies show a lower weight loss simply because they contain less free water.

## 4. Conclusions

High-performance mechanical applications do not tolerate a large scatter in compressive strength results within any given batch. This work sought to produce a high mechanical reliability binder matrix through the alkali activation of a blast-furnace slag using a sodium silicate solution, having not only high mechanical strength but also, low dispersion of the strength results. The use of a 3^3^ factorial Design of Experiments, with three factors (curing temperature, curing time, and slag particle size) in three levels, evidenced that, as expected, curing temperature and curing time were strongly correlated, being preponderant as control parameters to reach the best mechanical strength with the highest reliability (Weibull modulus). It was found that in short curing times (e.g., 24 h), alkali activation is not complete, while longer times promote both strength and reliability. Similarly, low-temperature cures (e.g., 30 °C) also fail to guarantee complete alkali activation, even for prolonged curing times. On the contrary, high curing temperatures (e.g., 90 °C) accelerate the residual water removal and originate structural defects that hinder mechanical strength and reliability.

The compromise between strength and mechanical reliability could be identified by overlapping the corresponding response surfaces, constructed in the factorial analysis: for the slag granulometries investigated, a curing temperature close to 60 °C and a curing time close to 40 h should ensure that a mean strength above 60 MPa is obtained with mechanical reliability of at least 5.0, for all investigated particle sizes. Moreover, this representation highlighted the subtle effect that particle size has on mechanical reliability by extending the working area: the precursor’s course granulometry, with a narrower particle size distribution, showed the best potential for high reliability.

For those curing conditions, the study of density variation during curing brought to light how particle size and size distribution first affect the initial suspension’s flowability and particle packing, and later, the final body’s density. As the processing parameters were kept constant, it could be observed that the lower viscosity of the suspension prepared with the broadest particle size distribution (d50 = 4 µm) prompted the entrapment of air bubbles during homogenization. Although a broad fine particle size distribution favors higher surface reactivity, hence, the alkali-activation reactions, it also favors lower viscosity and entrapped air bubbles, which harm both high strength and mechanical reliability. On the contrary, the course granulometry (d50 = 11 µm) with a narrower size distribution ensured enough matrix permeability for smooth water removal, likely due to its poorer particle packing, favoring its structural integrity. Despite the differences in particle sizes, under the selected curing conditions, no significant differences were observed in the extent of the alkali activation.

Indeed, the curing conditions were found to be particularly important for the water removal process. When the temperature and the time are mild, as in the pre-cure (24 h at 30 °C), the slower free water removal still enables the progress of the alkali activation. This was confirmed by the progressive intensity decrease in the characteristic FTIR spectroscopy bands, both for the reaction and water. These results were further confirmed by thermogravimetry. Although the matrix stiffness reached during pre-cure is already enough to oppose any further dimensional changes (shrinkage), as curing proceeds, it is insufficient to ensure the integrity of internal stresses induced by the continuous water removal. This explains the catastrophic consequences of aggressive cures.

Thus, the surface reactivity gained in the precursor’s mechanical activation (grinding) needs to be balanced by the role that the precursor’s granulometry plays in particle packing during processing, in the suspension stage (suspension’s viscosity and solids content), as well as in the drying stage (set body permeability and water removal, since dimensional changes that could relieve drying-induced stresses are impaired by early setting). In other words, the benefit gained from highly reactive finer particles can be quickly lost during processing. Knowledge of the role of the precursor’s granulometry, on the other hand, can also be used to improve processing, by adjusting (decreasing) the added water content and the homogenization speed, to ameliorate air entrapment.

## Figures and Tables

**Table 1:** A 3^3^ factorial Design of Experiments [37] for the AAB cure, using the granulometry, curing time, and curing temperature as factors.

Level	Granulometry	Curing Time (Hours)	Curing Temperature (°C)
1	100% B (B)	24	30
2	50% J + 50% B (J + B)	36	60
3	100% J (J)	48	90

**Table 2:** Chemical composition (wt% oxides, as determined by XRF) of blast-furnace slag.

Oxide	SiO_2_	Al_2_O_3_	CaO	MgO	K_2_O	MnO	SO_3_	SrO
Weight %	33.8	27.0	22.5	14.3	1.3	0.8	0.2	0.1

**Table 3:** Weibull mean strength (tmw) and mechanical reliability (Weibull modulus, m) for each configuration in the 3^3^ factorial Design of Experiments, using granulometry, curing time, and curing temperature as factors for the AAB cure.

Curing Temperature (°C)	Curing Time (Hours)	Granulometry	Weibull Mean Strength, tmw (MPa)	Mechanical Reliability, m	R^2^	Legend in Figure 3
30	24	J	**91.39 ± 17.67**	3.49	0.93	◼
		B	42.12 ± 18.80	1.86	0.97	◼
		J + B	**74.31 ± 18.72**	2.77	0.97	◼
	36	J	51.13 ± 10.73	3.75	0.98	◼
		B	49.92 ± 9.08	3.81	0.94	◼
		J + B	55.67 ± 7.93	4.27	0.89	◼
	48	J	56.31 ±7.64	**6.33**	0.91	◼
		B	13.04 ± 2.89	3.76	0.98	◼
		J + B	50.76 ± 6.41	**6.71**	0.92	◼
60	24	J	18.25 ± 5.03	3.16	0.96	◆
		B	56.44 ± 6.51	4.13	0.76	◆
		J + B	64.02 ± 8.62	3.84	0.82	◆
	36	J	60.84 ± 8.68	**5.71**	0.97	◆
		B	54.79 ± 6.25	**6.73**	0.97	◆
		J + B	55.60 ± 10.02	4.62	0.95	◆
	48	J	55.33 ±7.97	**5.21**	0.96	◆
		B	**78.95 ± 20.62**	3.44	0.97	◆
		J + B	**68.17 ± 13.92**	4.14	0.85	◆
90	24	J	51.02 ± 7.46	3.81	0.79	■
		B	42.83 ± 10.08	3.68	0.98	■
		J + B	56.53 ± 14.51	2.98	0.97	■
	36	J	**67.50 ± 13.07**	4.28	0.96	■
		B	36.34 ± 16.16	2.13	0.80	■
		J + B	45.14 ± 20.26	2.05	0.96	■
	48	J	65.73 ± 12.74	4.42	0.88	■
		B	35.69 ± 12.83	2.55	0.92	■
		J + B	51.77 ± 17.50	2.67	0.96	■

## Data Availability

The original contributions presented in the study are included in the article, further inquiries can be directed to the corresponding author.
